# Supplementary material for: Preferred auditory temporal processing regimes and auditory-motor synchronization
Source: Psychon Bull Rev. 2021 Jun 7;28(6):1860–73. doi: 10.3758/s13423-021-01933-w (PMC8642338; doi:10.3758/s13423-021-01933-w)
Supplement: Supplementary file 1 — (DOCX 41 kb) [file 13423_2021_1933_MOESM1_ESM.docx]

**Supplementary Material**

**S1. Table: Results of the Bayesian Model Comparison.**

| **i** | **Model Type** | **Onset High** | **Onset Low** | **Posterior Probability** | **Number of Iterations with Divergences** | **Number of Iterations that saturated the max treedepth** | **Number of chains with low BFMI** |
| --- | --- | --- | --- | --- | --- | --- | --- |
| 1 | Null | NA | NA | 9.35E-22 | 0 | 0 | 0 |
| 2 | MeanDiff | NA | NA | 2.15E-19 | 0 | 0 | 0 |
| 3 | Increase | 2 | 2 | 3.16E-07 | 0 | 0 | 0 |
| 4 | Increase | 2 | 3 | 2.00E-10 | 0 | 0 | 0 |
| 5 | Increase | 2 | 4 | 7.45E-14 | 0 | 0 | 0 |
| 6 | Increase | 2 | 5 | 2.84E-17 | 0 | 0 | 0 |
| 7 | Increase | 2 | 6 | 8.60E-20 | 0 | 0 | 0 |
| 8 | Increase | 2 | 7 | 9.92E-22 | 0 | 0 | 0 |
| 9 | Increase | 2 | 8 | 7.06E-23 | 0 | 0 | 0 |
| 10 | Increase | 3 | 2 | 0.00140196 | 0 | 0 | 0 |
| 11 | Increase | 3 | 3 | 5.76E-06 | 0 | 0 | 0 |
| 12 | Increase | 3 | 4 | 3.23E-09 | 0 | 0 | 0 |
| 13 | Increase | 3 | 5 | 3.45E-13 | 0 | 0 | 0 |
| 14 | Increase | 3 | 6 | 1.62E-16 | 0 | 0 | 0 |
| 15 | Increase | 3 | 7 | 1.84E-19 | 0 | 0 | 0 |
| 16 | Increase | 3 | 8 | 2.36E-21 | 0 | 0 | 0 |
| 17 | Increase | 4 | 2 | 0.069824313 | 0 | 0 | 0 |
| 18 | Increase | 4 | 3 | 0.005752703 | 0 | 0 | 0 |
| 19 | Increase | 4 | 4 | 2.40E-05 | 0 | 0 | 0 |
| 20 | Increase | 4 | 5 | 3.28E-09 | 0 | 0 | 0 |
| 21 | Increase | 4 | 6 | 6.32E-13 | 0 | 0 | 0 |
| 22 | Increase | 4 | 7 | 7.38E-17 | 0 | 0 | 0 |
| 23 | Increase | 4 | 8 | 1.17E-19 | 0 | 0 | 0 |
| 24 | Increase | 5 | 2 | 0.081328861 | 0 | 0 | 0 |
| 25 | Increase | 5 | 3 | 0.212079645 | 0 | 0 | 0 |
| 26 | Increase | 5 | 4 | 0.023375085 | 0 | 0 | 0 |
| 27 | Increase | 5 | 5 | 2.09E-05 | 0 | 0 | 0 |
| 28 | Increase | 5 | 6 | 7.42E-09 | 0 | 0 | 0 |
| 29 | Increase | 5 | 7 | 1.32E-13 | 0 | 0 | 0 |
| 30 | Increase | 5 | 8 | 1.40E-17 | 0 | 0 | 0 |
| 31 | Increase | 6 | 2 | 0.0010008 | 0 | 0 | 0 |
| 32 | Increase | 6 | 3 | 0.052748574 | 0 | 0 | 0 |
| 33 | Increase | 6 | 4 | 0.286141178 | 0 | 0 | 0 |
| 34 | Increase | 6 | 5 | 0.009498356 | 0 | 0 | 0 |
| 35 | Increase | 6 | 6 | 7.26E-05 | 0 | 0 | 0 |
| 36 | Increase | 6 | 7 | 1.04E-09 | 0 | 0 | 0 |
| 37 | Increase | 6 | 8 | 6.14E-15 | 0 | 0 | 0 |
| 38 | Increase | 7 | 2 | 8.25E-08 | 0 | 0 | 0 |
| 39 | Increase | 7 | 3 | 9.61E-06 | 0 | 0 | 0 |
| 40 | Increase | 7 | 4 | 0.000514935 | 0 | 0 | 0 |
| 41 | Increase | 7 | 5 | 0.000720913 | 0 | 0 | 0 |
| 42 | Increase | 7 | 6 | 0.000807573 | 0 | 0 | 0 |
| 43 | Increase | 7 | 7 | 3.46E-07 | 0 | 0 | 0 |
| 44 | Increase | 7 | 8 | 1.19E-12 | 0 | 0 | 0 |
| 45 | Increase | 8 | 2 | 4.77E-12 | 0 | 0 | 0 |
| 46 | Increase | 8 | 3 | 1.42E-10 | 0 | 0 | 0 |
| 47 | Increase | 8 | 4 | 3.44E-09 | 0 | 0 | 0 |
| 48 | Increase | 8 | 5 | 1.06E-08 | 0 | 0 | 0 |
| 49 | Increase | 8 | 6 | 1.81E-07 | 0 | 0 | 0 |
| 50 | Increase | 8 | 7 | 1.36E-08 | 0 | 0 | 0 |
| 51 | Increase | 8 | 8 | 4.42E-12 | 0 | 0 | 0 |
| 52 | IncreaseBaselineDiff | 2 | 2 | 4.75E-06 | 0 | 0 | 0 |
| 53 | IncreaseBaselineDiff | 2 | 3 | 1.25E-06 | 0 | 0 | 0 |
| 54 | IncreaseBaselineDiff | 2 | 4 | 2.31E-07 | 0 | 0 | 0 |
| 55 | IncreaseBaselineDiff | 2 | 5 | 3.44E-09 | 0 | 0 | 0 |
| 56 | IncreaseBaselineDiff | 2 | 6 | 7.41E-10 | 0 | 0 | 0 |
| 57 | IncreaseBaselineDiff | 2 | 7 | 2.94E-12 | 0 | 0 | 0 |
| 58 | IncreaseBaselineDiff | 2 | 8 | 9.43E-15 | 0 | 0 | 0 |
| 59 | IncreaseBaselineDiff | 3 | 2 | 8.93E-05 | 0 | 0 | 0 |
| 60 | IncreaseBaselineDiff | 3 | 3 | 3.99E-05 | 0 | 0 | 0 |
| 61 | IncreaseBaselineDiff | 3 | 4 | 1.01E-05 | 0 | 0 | 0 |
| 62 | IncreaseBaselineDiff | 3 | 5 | 1.72E-07 | 0 | 0 | 0 |
| 63 | IncreaseBaselineDiff | 3 | 6 | 3.72E-08 | 0 | 0 | 0 |
| 64 | IncreaseBaselineDiff | 3 | 7 | 1.22E-10 | 0 | 0 | 0 |
| 65 | IncreaseBaselineDiff | 3 | 8 | 2.81E-13 | 0 | 0 | 0 |
| 66 | IncreaseBaselineDiff | 4 | 2 | 0.000311192 | 0 | 0 | 0 |
| 67 | IncreaseBaselineDiff | 4 | 3 | 0.000253256 | 0 | 0 | 0 |
| 68 | IncreaseBaselineDiff | 4 | 4 | 0.000103671 | 0 | 0 | 0 |
| 69 | IncreaseBaselineDiff | 4 | 5 | 2.58E-06 | 0 | 0 | 0 |
| 70 | IncreaseBaselineDiff | 4 | 6 | 6.89E-07 | 0 | 0 | 0 |
| 71 | IncreaseBaselineDiff | 4 | 7 | 2.53E-09 | 0 | 0 | 0 |
| 72 | IncreaseBaselineDiff | 4 | 8 | 5.64E-12 | 0 | 0 | 0 |
| 73 | IncreaseBaselineDiff | 5 | 2 | 0.000780193 | 0 | 0 | 0 |
| 74 | IncreaseBaselineDiff | 5 | 3 | 0.001057597 | 0 | 0 | 0 |
| 75 | IncreaseBaselineDiff | 5 | 4 | 0.000669329 | 0 | 0 | 0 |
| 76 | IncreaseBaselineDiff | 5 | 5 | 2.59E-05 | 0 | 0 | 0 |
| 77 | IncreaseBaselineDiff | 5 | 6 | 9.22E-06 | 0 | 0 | 0 |
| 78 | IncreaseBaselineDiff | 5 | 7 | 4.21E-08 | 0 | 0 | 0 |
| 79 | IncreaseBaselineDiff | 5 | 8 | 1.05E-10 | 0 | 0 | 0 |
| 80 | IncreaseBaselineDiff | 6 | 2 | 0.001100381 | 0 | 0 | 0 |
| 81 | IncreaseBaselineDiff | 6 | 3 | 0.002356457 | 0 | 0 | 0 |
| 82 | IncreaseBaselineDiff | 6 | 4 | 0.002319161 | 0 | 0 | 0 |
| 83 | IncreaseBaselineDiff | 6 | 5 | 0.000143666 | 0 | 0 | 0 |
| 84 | IncreaseBaselineDiff | 6 | 6 | 7.12E-05 | 0 | 0 | 0 |
| 85 | IncreaseBaselineDiff | 6 | 7 | 4.58E-07 | 0 | 0 | 0 |
| 86 | IncreaseBaselineDiff | 6 | 8 | 1.46E-09 | 0 | 0 | 0 |
| 87 | IncreaseBaselineDiff | 7 | 2 | 2.34E-05 | 0 | 0 | 0 |
| 88 | IncreaseBaselineDiff | 7 | 3 | 8.34E-05 | 0 | 0 | 0 |
| 89 | IncreaseBaselineDiff | 7 | 4 | 0.000140983 | 0 | 0 | 0 |
| 90 | IncreaseBaselineDiff | 7 | 5 | 1.81E-05 | 0 | 0 | 0 |
| 91 | IncreaseBaselineDiff | 7 | 6 | 1.49E-05 | 0 | 0 | 0 |
| 92 | IncreaseBaselineDiff | 7 | 7 | 1.95E-07 | 0 | 0 | 0 |
| 93 | IncreaseBaselineDiff | 7 | 8 | 1.20E-09 | 0 | 0 | 0 |
| 94 | IncreaseBaselineDiff | 8 | 2 | 1.31E-09 | 0 | 0 | 0 |
| 95 | IncreaseBaselineDiff | 8 | 3 | 7.37E-09 | 0 | 0 | 0 |
| 96 | IncreaseBaselineDiff | 8 | 4 | 2.24E-08 | 0 | 0 | 0 |
| 97 | IncreaseBaselineDiff | 8 | 5 | 7.26E-09 | 0 | 0 | 0 |
| 98 | IncreaseBaselineDiff | 8 | 6 | 1.17E-08 | 0 | 0 | 0 |
| 99 | IncreaseBaselineDiff | 8 | 7 | 4.45E-10 | 0 | 0 | 0 |
| 100 | IncreaseBaselineDiff | 8 | 8 | 8.00E-12 | 0 | 0 | 0 |
| 101 | IncreaseSlopeDiff | 2 | 2 | 0.000287703 | 0 | 0 | 0 |
| 102 | IncreaseSlopeDiff | 2 | 3 | 0.000492144 | 0 | 0 | 0 |
| 103 | IncreaseSlopeDiff | 2 | 4 | 0.000291339 | 0 | 0 | 0 |
| 104 | IncreaseSlopeDiff | 2 | 5 | 3.16E-05 | 0 | 0 | 0 |
| 105 | IncreaseSlopeDiff | 2 | 6 | 3.20E-06 | 0 | 0 | 0 |
| 106 | IncreaseSlopeDiff | 2 | 7 | 8.90E-08 | 0 | 0 | 0 |
| 107 | IncreaseSlopeDiff | 2 | 8 | 2.95E-09 | 0 | 0 | 0 |
| 108 | IncreaseSlopeDiff | 3 | 2 | 0.002043928 | 0 | 0 | 0 |
| 109 | IncreaseSlopeDiff | 3 | 3 | 0.005264322 | 0 | 0 | 0 |
| 110 | IncreaseSlopeDiff | 3 | 4 | 0.004448363 | 0 | 0 | 0 |
| 111 | IncreaseSlopeDiff | 3 | 5 | 0.000519583 | 0 | 0 | 0 |
| 112 | IncreaseSlopeDiff | 3 | 6 | 5.13E-05 | 0 | 0 | 0 |
| 113 | IncreaseSlopeDiff | 3 | 7 | 9.86E-07 | 0 | 0 | 0 |
| 114 | IncreaseSlopeDiff | 3 | 8 | 2.05E-08 | 0 | 0 | 0 |
| 115 | IncreaseSlopeDiff | 4 | 2 | 0.002379171 | 0 | 0 | 0 |
| 116 | IncreaseSlopeDiff | 4 | 3 | 0.011504829 | 0 | 0 | 0 |
| 117 | IncreaseSlopeDiff | 4 | 4 | 0.019075127 | 0 | 0 | 0 |
| 118 | IncreaseSlopeDiff | 4 | 5 | 0.003054384 | 0 | 0 | 0 |
| 119 | IncreaseSlopeDiff | 4 | 6 | 0.000372851 | 0 | 0 | 0 |
| 120 | IncreaseSlopeDiff | 4 | 7 | 4.70E-06 | 0 | 0 | 0 |
| 121 | IncreaseSlopeDiff | 4 | 8 | 5.22E-08 | 0 | 0 | 0 |
| 122 | IncreaseSlopeDiff | 5 | 2 | 0.000780758 | 0 | 0 | 0 |
| 123 | IncreaseSlopeDiff | 5 | 3 | 0.009639493 | 0 | 0 | 0 |
| 124 | IncreaseSlopeDiff | 5 | 4 | 0.053083492 | 0 | 0 | 0 |
| 125 | IncreaseSlopeDiff | 5 | 5 | 0.019922897 | 0 | 0 | 0 |
| 126 | IncreaseSlopeDiff | 5 | 6 | 0.005310045 | 0 | 0 | 0 |
| 127 | IncreaseSlopeDiff | 5 | 7 | 4.87E-05 | 0 | 0 | 0 |
| 128 | IncreaseSlopeDiff | 5 | 8 | 2.26E-07 | 0 | 0 | 0 |
| 129 | IncreaseSlopeDiff | 6 | 2 | 1.66E-05 | 0 | 0 | 0 |
| 130 | IncreaseSlopeDiff | 6 | 3 | 0.000535788 | 0 | 0 | 0 |
| 131 | IncreaseSlopeDiff | 6 | 4 | 0.01462935 | 0 | 0 | 0 |
| 132 | IncreaseSlopeDiff | 6 | 5 | 0.028905509 | 0 | 0 | 0 |
| 133 | IncreaseSlopeDiff | 6 | 6 | 0.059605619 | 0 | 0 | 0 |
| 134 | IncreaseSlopeDiff | 6 | 7 | 0.000866154 | 0 | 0 | 0 |
| 135 | IncreaseSlopeDiff | 6 | 8 | 1.59E-06 | 0 | 0 | 0 |
| 136 | IncreaseSlopeDiff | 7 | 2 | 3.44E-09 | 0 | 0 | 0 |
| 137 | IncreaseSlopeDiff | 7 | 3 | 1.11E-07 | 0 | 0 | 0 |
| 138 | IncreaseSlopeDiff | 7 | 4 | 5.70E-06 | 0 | 0 | 0 |
| 139 | IncreaseSlopeDiff | 7 | 5 | 4.69E-05 | 0 | 0 | 0 |
| 140 | IncreaseSlopeDiff | 7 | 6 | 0.001596641 | 0 | 0 | 0 |
| 141 | IncreaseSlopeDiff | 7 | 7 | 0.000217413 | 0 | 0 | 0 |
| 142 | IncreaseSlopeDiff | 7 | 8 | 5.37E-07 | 0 | 0 | 0 |
| 143 | IncreaseSlopeDiff | 8 | 2 | 2.05E-13 | 0 | 0 | 0 |
| 144 | IncreaseSlopeDiff | 8 | 3 | 2.26E-12 | 0 | 0 | 0 |
| 145 | IncreaseSlopeDiff | 8 | 4 | 3.51E-11 | 0 | 0 | 0 |
| 146 | IncreaseSlopeDiff | 8 | 5 | 1.58E-10 | 0 | 0 | 0 |
| 147 | IncreaseSlopeDiff | 8 | 6 | 9.73E-09 | 0 | 0 | 0 |
| 148 | IncreaseSlopeDiff | 8 | 7 | 1.84E-08 | 0 | 0 | 0 |
| 149 | IncreaseSlopeDiff | 8 | 8 | 1.01E-09 | 0 | 0 | 0 |

**S2. Table**: Results of the WUD procedure at each standard rate (in Hz and cycle duration in ms) expressed as the relative fraction (in %) of the oscillatory cycle in ms, x = (fraction_standard_ – fraction_comparison_) / fraction_standard_ * 100, required for reliable rate discrimination. (SD: standard deviation, Min: minimum, Max: maximum, Mdn: median, IQR: inter-quartile range)

|  | 4 Hz (250ms) | 5.6Hz  (178.6ms) | 7.1 Hz  (140ms) | 8.7 Hz  (114.8ms) | 10.3 Hz  (97.2ms) | 11.8 Hz  (84.3ms) | 13.4 Hz  (74.5ms) | 15 Hz  (66.7ms) |
| --- | --- | --- | --- | --- | --- | --- | --- | --- |
| Mean | 4.65 | 4.57 | 5.06 | 6.19 | 5.82 | 7.19 | 8.44 | 11.48 |
| SD | 2.62 | 2.62 | 2.83 | 4.24 | 3.28 | 4.38 | 4.81 | 7.42 |
| Min | 1.53 | 1.28 | 1.19 | 1.28 | 0.85 | 1.74 | 2.48 | 2.08 |
| Max | 11.54 | 13.89 | 14.41 | 22.15 | 14.29 | 22.76 | 20.87 | 31.74 |
| Mdn | 3.92 | 4.12 | 4.38 | 4.95 | 4.55 | 5.7 | 7.66 | 9.5 |
| IQR | 3.12 | 2.47 | 2.36 | 4.43 | 4.08 | 5.28 | 6.61 | 8.38 |

**S3. Table**: Results of the CS procedure at each standard rate (in Hz and cycle duration in ms) expressed as the relative fraction (in %) of the oscillatory cycle in ms, x = (fraction_standard_ – fraction_comparison_) / fraction_standard_ * 100, required for reliable rate discrimination. (SD: standard deviation, Min: minimum, Max: maximum, Mdn: median, IQR: inter-quartile range)

|  | 4 Hz (250ms) | 11.8 Hz  (84.3ms) |
| --- | --- | --- |
| Mean | 3.54 | 5.39 |
| SD | 1.77 | 2.93 |
| Min | 1.19 | 1.23 |
| Max | 8.47 | 16.19 |
| Mdn | 3.03 | 4.62 |
| IQR | 2.55 | 2.32 |
